# Comprehensive dataset of dynamic probing heavy test results for subsurface characterization^☆^

**DOI:** 10.1016/j.dib.2025.111391

**Published:** 2025-02-19

**Authors:** E. Soranzo

**Affiliations:** Department of Civil Engineering and Natural Hazards, Peter Jordan Straße 82, Vienna, 1190, Austria

**Keywords:** Dynamic probing heavy, Geotechnical engineering, In-situ testing, Soil characterization, Soil properties, Subsurface investigation, Subsurface profiling

## Abstract

This dataset presents comprehensive results from Dynamic Probing Heavy tests conducted for subsurface characterization. The Dynamic Probing Heavy test is a widely used and standardized in-situ testing method for assessing soil properties, particularly in geotechnical engineering. The dataset includes measurements of the number of blows per 10 cm penetration and corresponding depths for various locations and projects. The data were collected from multiple test sites, providing a robust and diverse set of subsurface profiles. Each test result is presented in a standardized format, ensuring consistency and ease of use for further analysis. The dataset is valuable for researchers and practitioners in geotechnical engineering, giving information about soil behaviour and aiding in the design and analysis of building foundations, earthworks and other geotechnical structures. Although the data were collected exclusively from test sites in Austria and may thus not fully represent all soil types and conditions and the precision of the blow count measurements is affected by the accuracy of the operators, the dataset can be used for clustering and synthetic data generation, contributing to the advancement of geotechnical knowledge and practice.

## Highlights

• The dataset provides comprehensive results from Dynamic Probing Heavy (DPH) tests in Austria.
• Detailed measurements of depth (z) and blows per 10 cm penetration ($N_{10}$) are included.
• The dataset is standardized and includes 409 CSV files with 97,345 measurements for consistency.
• Researchers can use this dataset to derive mechanical soil parameters for geotechnical design.
• The data can be post-processed using statistical and machine learning methods, including clustering.

Specification Table

**Table:** 

Subject	Geotechnical Engineering
Specific subject area	Soil Characterization
Type of data	CSV files
Data collection	In-situ DPH tests
Data-source location	Austria
Data accessibility	[6]
Related research articles	None

## 1 Value of the Data

• These data are useful in understanding the subsurface characteristics and soil properties at various locations, which are critical for geotechnical engineering applications.
• Researchers, geotechnical engineers and practitioners can benefit from this dataset through its detailed measurements of depth (z) and the number of blows per 10 cm penetration ($N_{10}$), which give information about soil resistance and stratification.
• The dataset can be applied in various real-world scenarios, such as urban development projects, infrastructure planning and geotechnical engineering studies. For instance, it can be used to assess soil conditions for foundation design, evaluate slope stability and plan underground utilities.
• The dataset can be reused for deriving mechanical soil parameters such as the density index, friction angle and oedometric modulus required for the design and analysis of building foundations, earthworks and other geotechnical structures.
• The depth measurements help in identifying different soil layers and their interfaces, which is crucial for understanding the subsurface profile and planning construction activities accordingly.
• The data can be post-processed using various statistical and machine learning methods, including clustering to define the soil layers at the project locations and to generate synthetic test data.
• The dataset includes results from multiple test sites and projects, ensuring a diverse set of subsurface profiles. This diversity enhances the dataset’s applicability across different geotechnical scenarios and soil types.
• The dataset’s standardized CSV format ensures easy integration with other datasets like CPT data and borehole logs. This facilitates comprehensive subsurface characterization and enhances the accuracy of geotechnical models. Additionally, the dataset can be used in GIS applications for improved soil property prediction and spatial analysis, given that most data is named after the collection location.
• By making this dataset publicly available, we aim to foster collaboration and knowledge sharing among researchers and practitioners in the geotechnical engineering community, promoting transparency and reproducibility in research.

## 2 Background

The dataset was collected by the Laboratory of Geotechnical Engineering at BOKU University during fieldwork conducted for various clients, including building companies, state-owned enterprises, federal states and municipalities. The primary motivation behind compiling this dataset was to survey the soil in terms of its bearing capacity, which is crucial for infrastructure development and construction projects.

DPH tests are a widely used and standardized in-situ testing method in geotechnical engineering [5]. These tests involve driving a standardized probe into the ground and recording the number of blows required to penetrate each 10 cm interval. The resulting data give information about the subsurface conditions, particularly soil resistance, stiffness and stratification. Some correlations between $N_{10}$ and the density index, soil friction angle and oedometric modulus are given in the standards [4] and reported in Section 3. This information is essential for the design and analysis of foundations, earthworks and other geotechnical structures.

## 3 Data Description

**Fig. 1:**
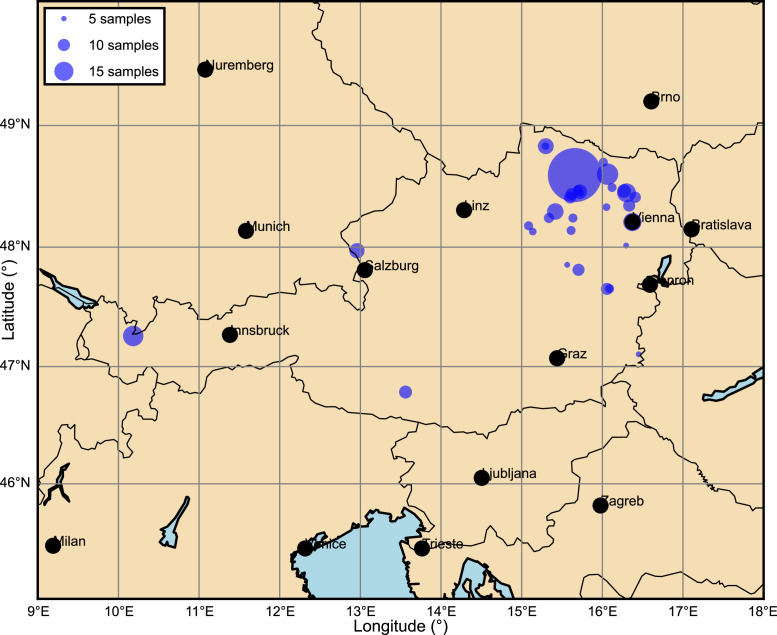
Geographical distribution of the Dynamic Probing Heavy (DPH) field test locations across Austria. Each circle represents a test site where multiple DPH tests were conducted.

There are 409 CSV files in the dataset. File naming follows this structure: Project_ID_Location.csv. Various DPH tests (ID) are conducted within the same test campaign (Project) at a certain location (Location). Table 1 summarizes the key data characteristics, grouping the tests per location. The test locations and their numerosity are depicted in Fig. 1. The CSV files comprise the following columns:

• id Identification number of the test (same as ID in the file name)
• i1 Record number (continuous numbering of each data row)
• z Depth in meters in 0.1 m steps
• n10 Number of blows necessary for the probe to penetrate 10 cm into the soil

**Table 1:** Summary of key data characteristics by location.

Location	Total no.	Mean no.	Median no.	Min depth	Max depth	Max blow
	of tests	of tests	of tests			count
	(-)	(-)	(-)	(m)	(m)	(-)
Aggsbach Dorf	755	62.9	79	8.6	1	128
Alleinsteig	92	23.0	24.5	3.6	0	40
Allentsteig	30	30.0	30	3.0	1	15
Birngruber	58	19.3	19	2.0	1	11
Diendorf am Kamp	100	50.0	50	5.0	0	11
Egelsee	96	13.7	12	2.5	6	25
Emmersdorf	90	22.5	21.5	3.8	1	153
Gars am Kamp	10572	80.7	80	10.0	0	135
Gneixendorf	108	18.0	18	1.8	1	15
Göllersdorf	210	70.0	70	7.0	0	19
Hadersdorf am Kamp	82	13.7	13.5	1.7	0	10
Haßbach	55	9.2	8.5	1.5	3	58
Kirchau	42	14.0	14	1.5	2	23
Kleineberharts	62	31.0	31	4.6	1	100
Korneuburg	112	18.7	17.5	2.5	2	18
Krems	284	47.3	45	9.5	0	140
Kulm	10	10.0	10	1.0	4	15
Mollmannsdorf	177	35.4	35	4.1	3	14
Niederhollabrunn	317	19.8	16	3.0	0	24
Oberarnsdorf	430	61.4	60	7.0	0	65
Oberhauzental	40	40.0	40	4.0	3	17
Pernersdorf	40	13.3	15	1.5	6	34
Ragelsdorf	26	8.7	7	1.3	7	70
Schongräbern	274	13.0	12	2.1	1	60
Schwarzau	250	41.7	50	5.0	0	118
St. Aegyd	21	21.0	21	2.1	16	81
St. Georgen	123	41.0	41	4.1	1	10
Streitdorf	119	14.9	15	1.6	1	8
Traiskirchen	21	21.0	21	2.1	1	48
Tulln	18	9.0	9	0.9	10	145
Unknown/None	101004	1202.4	30.5	15.2	0	300
Vestenötting	322	35.8	31	6.0	1	100
Vienna	602	43	37	8	0	300
Warth	213	11.2	11	1.8	1	31
Wieselburg	59	29.5	29.5	3.0	5	44

In total, the dataset contains 97,345 measurements. The z values, representing depth, have a mean of approximately 3.57 m with a standard deviation of 2.64, indicating moderate variability. Depths range from 0.1 to 15.2 m, with half of the values below 3.0 m. Most measurements are thus from shallow to moderate depths.

The $N_{10}$ values, representing blow counts, have a mean of 14.25 and a higher variability with a standard deviation of 16.38. Blow counts range from 0 to 300, with half of the values below 8, indicating that the majority of measurements are in soft to moderately dense soils [4]. The maximum blow count of 300 suggests the presence of outliers or very dense materials. DPH tests are typically stopped when reaching 100 blows, as per standard practice [5]. However, in some cases, tests were prolonged beyond this limit. These extended tests are primarily found in Vienna, indicating inconsistencies in adhering to the stopping criteria.

The reasons for prolonging the tests beyond the standard limit are not entirely clear. It is possible that the operators continued the tests to gather more data on particularly dense soil layers, or there may have been inconsistencies in following the stopping criteria. As a result, these high blow count values could indicate either genuinely dense soil conditions or artifacts introduced during data collection.

**Fig. 2:**
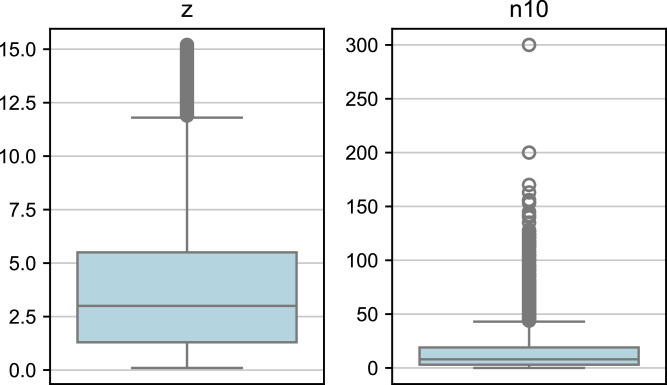
Boxplots showing the descriptive statistics of the dataset. The left boxplot represents the depth (z) values in meters, while the right boxplot represents the blow count ($N_{10}$) values. The boxplots illustrate the median, quartiles and potential outliers in the dataset.

**Fig. 3:**
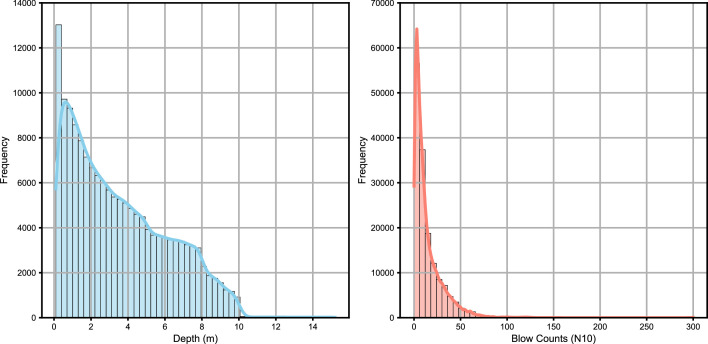
Histograms showing the distribution of depth (z) and blow counts ($N_{10}$) in the dataset. The left histogram represents the depth distribution, while the right histogram represents the blow count distribution. Both histograms include kernel density estimates (KDE) and grid lines to enhance interpretability.

The close alignment of the median and mean for z indicates a balanced distribution, while the higher variability in $N_{10}$ reflects the diversity in soil resistance across the sampled locations (Fig. 2). The data shows positive skew for both depth and blow counts, with a few high values extending the ranges (Fig. 3).

A correlation analysis between depth and blow counts was performed, revealing a moderate positive correlation with a coefficient of 0.35. This indicates that blow counts tend to increase with depth, suggesting the presence of denser soil layers at greater depths.

## 4 Experimental Design, Materials and Methods

**Fig. 4:**
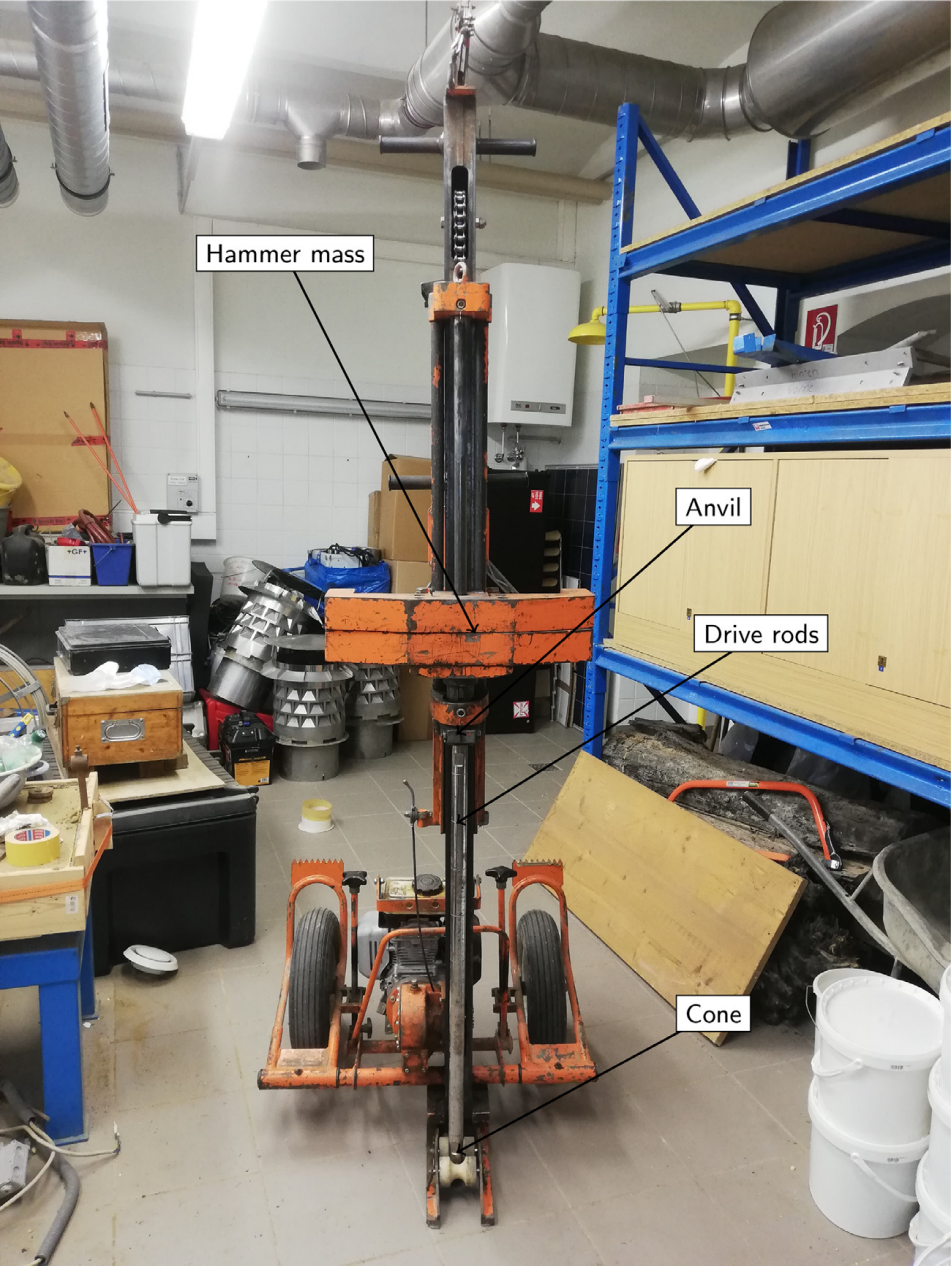
DPH testing equipment of the Institute of Geotechnical Engineering at BOKU.

The DPH machine of the Institute of Geotechnical Engineering at BOKU University is shown in Fig. 4. The experimental design and methods for acquiring data with the DPH equipment are detailed as follows according to the standards [5]. A steel hammer is used in the driving device. An automatic release mechanism ensures a constant free fall, with negligible speed of the hammer when released and no induced parasitic movements in the drive rods. The hammer mass for DPH is specified as 50 kg with a height of fall of 500 mm to 1000 mm. The anvil diameter is between 50 mm and 185 mm, with a maximum mass of 30 kg. The nominal base area of the cone is 43.7 cm${}^{2}$, with a base diameter of 42 mm when new and a maximum permissible wear of 1 mm. The drive rods have a maximum mass of 3 kg/m and a diameter of 32 mm, with an axial deviation not exceeding 1 mm/m.

Dynamic probing is generally performed from the ground surface and the number of blows $N_{10}$ is recorded continuously. The penetrometer is set up vertically, ensuring no displacement during testing. The inclination of the driving mechanism and the driving rod projecting from the ground does not deviate by more than 2% from the vertical, with allowances up to 5% in difficult ground conditions.

The penetrometer is continuously driven into the ground at a driving rate between 15 and 30 blows per minute, except in sand and gravel where the rate may be increased up to 60 blows per minute. The rods are rotated by 1.5 turns at least every 1.0 m.

The number of blows $N_{10}$ is recorded as a function of depth. The results are generally presented as a step diagram with the dynamic probing results on the horizontal axis and depth on the vertical axis.

The machine was produced by the company Magdeburger Prüfgerätebau and has the following dimensions (H×B×L): 2370×790×780 mm. The weight amounts to about 110 kg (without steel hammer) and the number of hammer blows are within the range 15–30 blows/min.

Calibration of the DPH equipment was performed regularly according to the standards [5]. This included dimensional checks before each test, rod straightness at each new site and at least every 20 tests at the same site, hammer drop height, friction-free fall of the hammer, condition of the anvil and release mechanism, energy measurement per blow every six months. To mitigate human error, operators followed the testing protocols outlined in the standards. The presence of two operators, required to handle the equipment (loading, unloading and transport), ensured measuring consistency as two independent blow counts were conducted.

In addition to regular calibration and operator training with mutual checks, methods for identifying potential errors or inconsistencies include considering geotechnical influences. The penetration resistance is influenced by factors such as soil type, grain size distribution, grain shape, roughness, mineral type, degree of compaction and stress conditions. For example, coarse-grained soils with sharp or rough grains exhibit higher penetration resistance compared to soils with rounded and smooth grains. The presence of stones and blocks can significantly increase resistance. The blow count in coarse-grained soils below the groundwater table is generally lower due to reduced effective vertical stress. In fine-grained soils, the blow count may remain the same or increase due to capillary action. Additionally, the penetration resistance increases with depth until a critical depth is reached, beyond which it remains relatively constant [5].

Correlations exist in the literature between the number of blows of the DPH and various geotechnical parameters [4]. The density index $I_{D}$ can be obtained as [7]:

(1) $$I_{D}=0.10+0.435\mathrm{lg}N_{10}$$

This correlation is valid for poorly-graded sand with uniformity coefficient $C_{U}\leq 3$ above groundwater and a blow count $3\leq N_{10}\leq 50$, whereas below groundwater the following correlation can be considered:

(2) $$I_{D}=0.23+0.380\mathrm{lg}N_{10}$$

For well-graded sand-gravel with $C_{U}\geq 6$ above groundwater the correlation

(3) $$I_{D}=-0.14+0.550\mathrm{lg}N_{10}$$

can be considered.

The effective angle of shearing resistance ($\varphi^{\prime }$) can then be derived from the density index ($I_{D}$) according to Table 2 from [2].

**Table 2:** Effective angle of shearing resistance of coarse soil $\varphi^{\prime }$ as function of the density index $I_{D}$ and the uniformity coefficient $C_{U}$.

Soil type	Grading	Range of $I_{D}$	Effective angle of shearing resistance $\varphi^{\prime }$
		(%)	(${}^{\circ }$)
Slightly fine-grained sand	$C_{U}<6$	15–35	30
Sand, sand-gravel		35–65	32.5
		> 65	35
Sand, sand-gravel	$6\leq C_{U}\leq 15$	15–35	30
gravel		35–65	34
		> 65	38

The vertical stress dependent oedometer settlement modulus ($E_{\mathrm{oed}}$), frequently recommended for settlement calculation of spread foundations, can be defined as follows [1], [3], [7]:

(4) $$E_{\mathrm{oed}}=w_{1}p_{a}{\left(\frac{\sigma_{v}^{\prime }+0.5\Delta \sigma_{v}^{\prime }}{p_{a}}\right)}^{w_{2}}$$

where

• $w_{1}$ is the stiffness coefficient;
• $w_{2}$ is the stiffness exponent. For sands with a uniformity coefficient $C_{U}\leq 3$; 3: $w_{2}=0.5$; for clays of low plasticity ($I_{p}\leq 10$; $w_{L}\leq 35$): $w_{2}=0.6$;
• $\sigma_{v}^{\prime }$ is the effective vertical stress at the base of the foundation or at any depth below it due to overburden of the soil;
• $\Delta \sigma_{v}^{\prime }$ is the effective vertical stress caused by the structure at the base of the foundation or at any depth below it;
• $p_{a}$ is the atmospheric pressure;
• $I_{p}$ is the plasticity index;
• $w_{L}$ is the liquid limit.

Values for the stiffness coefficient ($w_{1}$) can be derived from DPH tests using the following equations, depending on the soil type. For poorly-graded sands ($C_{U}\leq 3$) above groundwater

(5) $$w_{1}=249\mathrm{lg}N_{10}+161$$

with range of validity $3\leq N_{10}\leq 10$. For low-plasticity clays of at least stiff consistency ($0.75\leq I_{C}\leq 1.30$) above groundwater:

(6) $$w_{1}=6N_{10}+50$$

with range of validity $3\leq N_{10}\leq 13$.

## Limitations

The dataset has the following limitations:

1. **Geographical limitation**: the data for this study were collected exclusively from test sites in Austria, which inherently limits the direct applicability of the findings to other geographical regions with different soil characteristics. Austria’s soil conditions, influenced by its unique geological history and climatic conditions, may not be representative of soils found in other parts of the world. For instance, regions with tropical climates, arid deserts or areas with significant volcanic activity may exhibit soil properties that differ significantly from those observed in Austria.
2. **Data homogeneity**: since the dataset used in this study is derived from specific projects and locations, it may not encompass the full spectrum of soil types and conditions present even within Austria. This potential lack of data diversity could limit the generalizability of the findings to other areas with different soil compositions. To enhance the generalizability of the findings, future studies should aim to include a more diverse range of soil types and conditions by expanding the geographical scope of data collection and incorporating a variety of project types and locations.
3. **Measurement precision**: The precision of the blow count measurements is affected by the accuracy of the operators, potentially introducing minor errors in the data.

## Ethics Statement

The author has read and follow the ethical requirements for publication in Data in Brief and confirms that the current work does not involve human subjects, animal experiments, or any data collected from social media platforms.

## Declaration of generative AI and AI-assisted technologies in the writing process

During the preparation of this work the author used ChatGPT in order to improve readability and language of the work under human oversight and control and after careful review and edit of the result to avoid authoritative-sounding output that can be incorrect, incomplete or biased. After using this tool/service, the author(s) reviewed and edited the content as needed and take(s) full responsibility for the content of the published article.

## CRediT authorship contribution statement

**E. Soranzo:** Conceptualization, Data curation, Formal analysis, Funding acquisition, Methodology, Project administration, Software, Supervision, Validation, Visualization, Writing – original draft, Writing – review & editing.
